# Quantitative microscopy reveals dynamics and fate of clustered IRE1α

**DOI:** 10.1073/pnas.1915311117

**Published:** 2019-12-23

**Authors:** Vladislav Belyy, Ngoc-Han Tran, Peter Walter

**Affiliations:** ^a^Howard Hughes Medical Institute, University of California, San Francisco, CA 94158;; ^b^Department of Biochemistry and Biophysics, University of California, San Francisco, CA 94158

**Keywords:** unfolded protein response, IRE1, clustering, signaling, quantitative microscopy

## Abstract

The endoplasmic reticulum (ER) membrane-resident stress sensor inositol-requiring enzyme 1 (IRE1) governs the most evolutionarily conserved branch of the unfolded protein response. Upon sensing an accumulation of unfolded proteins in the ER lumen, IRE1 activates its cytoplasmic kinase and ribonuclease domains to transduce the signal. IRE1 activity correlates with its assembly into large clusters, yet the biophysical characteristics of IRE1 clusters remain poorly characterized. We combined superresolution microscopy, single-particle tracking, fluorescence recovery, and photoconversion to examine IRE1 clustering quantitatively in living human and mouse cells. Our results revealed that: 1) In contrast to qualitative impressions gleaned from microscopic images, IRE1 clusters comprise only a small fraction (∼5%) of the total IRE1 in the cell; 2) IRE1 clusters have complex topologies that display features of higher-order organization; 3) IRE1 clusters contain a diffusionally constrained core, indicating that they are not phase-separated liquid condensates; 4) IRE1 molecules in clusters remain diffusionally accessible to the free pool of IRE1 molecules in the general ER network; 5) when IRE1 clusters disappear at later time points of ER stress as IRE1 signaling attenuates, their constituent molecules are released back into the ER network and not degraded; 6) IRE1 cluster assembly and disassembly are mechanistically distinct; and 7) IRE1 clusters’ mobility is nearly independent of cluster size. Taken together, these insights define the clusters as dynamic assemblies with unique properties. The analysis tools developed for this study will be widely applicable to investigations of clustering behaviors in other signaling proteins.

## Significance

The endoplasmic reticulum (ER) is the site for folding and maturation of secreted and membrane proteins. When the ER protein-folding machinery is overwhelmed, misfolded proteins trigger ER stress, which is frequently linked to human diseases, including cancer and neurodegeneration. Inositol-requiring enzyme 1 (IRE1) is an ER membrane-resident sensor that assembles into large clusters of previously unknown organization upon its activation by unfolded peptides. We demonstrate that IRE1 clusters are topologically complex dynamic structures that remain contiguous with the ER membrane throughout their lifetime. The majority of clustered IRE1 molecules are diffusionally trapped inside the clusters until IRE1 signaling attenuates, at which point they are released back into the ER through a pathway that is functionally distinct from cluster assembly.

The ability to sense and respond to cellular stresses, such as the accumulation of misfolded proteins in the endoplasmic reticulum (ER), is essential for maintaining homeostasis in eukaryotic cells (1). A conserved set of signaling pathways, collectively termed the unfolded protein response (UPR), allows cells to sense ER stress and reestablish ER homeostasis through the coordinated actions of transcriptional and translational regulatory networks (2–6). Of the 3 branches of the UPR, the best-studied and most-conserved is governed by the ER-resident transmembrane kinase/ribonuclease (RNase) inositol-requiring enzyme 1 (IRE1) (7–9). Because the UPR is implicated in many cellular processes and human diseases (4), including cancer, metabolic syndromes, and neurodegeneration, the mechanistic details behind IRE1 signaling are of outstanding interest.

IRE1 consists of an ER lumenal domain, a single transmembrane helix followed by a cytosolic flexible linker, a kinase, and a C-terminal RNase domain (9). Of the 2 isoforms present in human, IRE1α and IRE1β (10, 11), IRE1α is ubiquitously expressed across most cell types and tissues (12) and is the focus of this study. During ER stress, IRE1 binds to unfolded proteins via ligand interactions with its lumenal domain (13–15). This drives activation by cooperative oligomerization, *trans*-autophosphorylation (16), and subsequent allosteric activation of its RNase domain (17). Active IRE1 cleaves its mRNA substrates (18) (*Hac1* mRNA in yeast and *XBP1* mRNA in metazoans) and initiates a noncanonical splicing reaction independent of the spliceosome (9), a critical step in generating active Hac1s/XBP1s transcription factors (19). Hac1s/XBP1s potently up-regulate several hundred genes that serve to reestablish ER proteostasis (20).

Oligomerization is central to IRE1’s activation both in cells (21) and in vitro (17). IRE1 forms distinct clusters upon ER stress in both yeast and metazoan cells (21, 22), and IRE1 lumenal and cytosolic domains were crystalized as both dimers (23) and higher oligomers (14, 17). In addition, disruptions of lumenal as well as cytosolic oligomerization interfaces were shown to simultaneously abolish clustering and diminish IRE1’s RNase activity (17, 21, 24, 25). However, despite this wealth of evidence pointing to the significance of clustering, many basic questions regarding the biophysical nature of IRE1 clusters remain unanswered.

First, it remains unknown whether IRE1 molecules in clusters are locked in place within a solid-like structure or can freely diffuse inside the cluster and across the cluster boundary. Ordered arrangements of IRE1 oligomers observed in crystal structures are more consistent with the former possibility, while reports that clusters grow via fusion (26) favor the latter. Second, the fate of clustered IRE1 following ER stress remains a subject of debate. Clusters eventually disappear when cells are challenged with prolonged ER stress, concomitantly with attenuation of IRE1’s RNase activity. The clearance of clusters could be achieved by regulated degradation—making IRE1 activation a 1-way pathway akin to many receptor tyrosine kinases (27)—or the clustered proteins could be recycled back into the ER membrane via a mechanism that precludes immediate reactivation (21). Finally, many other supramolecular signaling clusters rely on close associations with cytoskeletal filaments (28), and IRE1 itself has been postulated to interact physically with the cytoskeleton and closely associate with various organelles (29, 30). However, it remains unclear whether these interactions hold true for the clustered form of IRE1 and whether the clustering phenomenon may be regulated by interorganelle contacts. Intriguingly, these questions highlight close parallels between the study of ER membrane sensors and the broad, rapidly evolving fields of plasma membrane receptor signaling and phase separation in cellular signaling cascades (28, 31, 32).

In this study, we address the above questions and develop a general analysis toolbox for reproducible and unbiased experimental quantification of ER receptor clustering. Pharmacological manipulation of IRE1 has shown that clusters can exhibit fundamentally different properties depending on what molecular trigger drives their formation (26, 33). Thus, a careful analysis of IRE1’s clustering behavior provides a unique window into its underlying mechanism of activation.

## Results

### Morphological Complexity of IRE1 Clusters.

**Fig. 1.**
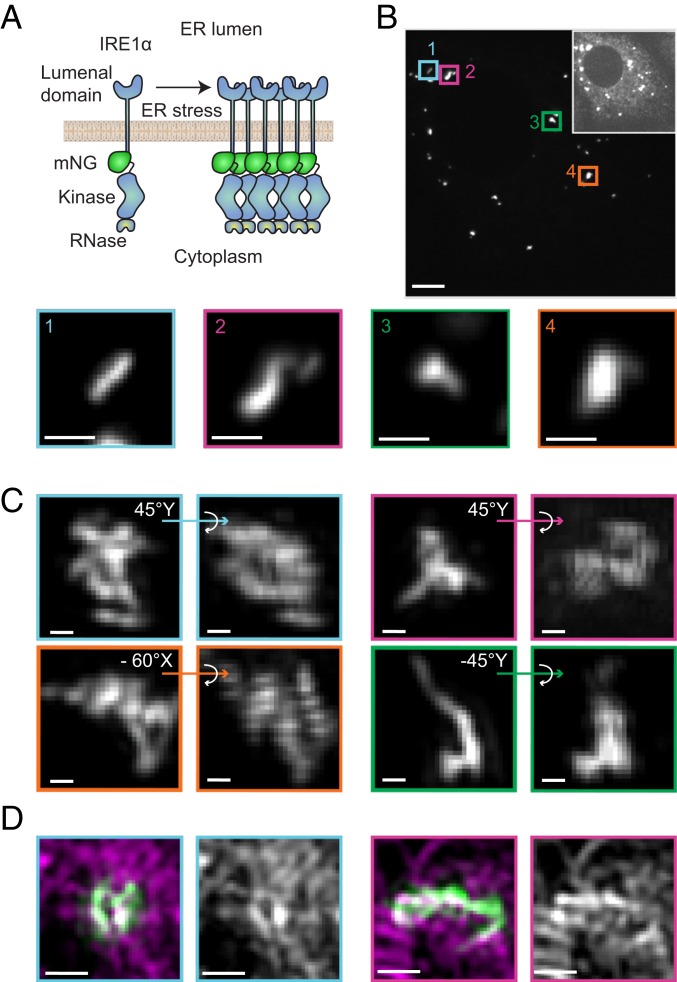
Diverse morphologies of IRE1 clusters. (*A*) Schematic depiction of the clustering assay. IRE1-mNG molecules assemble into large clusters in the plane of the membrane when treated with an ER stress agent. (*B*, *Top*) Maximum intensity projection spinning-disk confocal image of live MEF-IRE1-mNG cells in mNeonGreen channel. Oversaturated *Inset* highlights cell shape. (Scale bar, 5 μm.) (*Bottom*) Magnified bilinear interpolation of selected images of interesting cluster morphologies. (Scale bars, 1 μm.) (*C*) Deconvoluted SIM images of IRE1-mNG clusters in fixed and stressed (4 h Tm) MEF-IRE1-mNG cells. For each magnified large cluster, bilinear interpolation of the maximum-intensity projection together with indicated 3D projection is shown to highlight topological complexities. (Scale bars, 200 nm.) (*D*) Representative SIM bilinear interpolation images showing: (*First Left* and *First Right*) overlay of IRE1-mNG in green and HaloTag-Sec61β labeled with the HaloTag-JF_549_ dye in magenta, and (*Second Left* and *Second Right*) HaloTag-Sec61β signal representing local ER structure. (Scale bars, 500 nm.) Full cells and additional SIM examples are found in *SI Appendix*, Fig. S2.

To gain insight into the structural organization of clustered IRE1, we obtained high-resolution images of the clusters. To circumvent the photobleaching and low signal-to-noise ratios inherent to previously published IRE1-GFP constructs, we fused IRE1 to the exceptionally bright mNeonGreen (mNG) fluorescent protein (34) (Fig. 1*A*). This IRE1α construct (IRE1-mNG) includes a tandem FLAG tag, a His6 tag, and mNG inserted in the cytoplasmic juxtamembrane linker region. We expressed IRE1-mNG under the control of a tetracycline-inducible promoter (*SI Appendix*, Fig. S1) in mouse embryonic fibroblasts (MEFs) isolated from IRE1α/IRE1β knockout (KO) mice (25) by lentiviral integration to generate a stable MEF cell line expressing IRE1-mNG (MEFs-IRE1-mNG).

Upon doxycycline induction, IRE1-mNG was expressed and exhibited a characteristic reticulated distribution indicative of ER localization. In agreement with previous findings, we observe that IRE1-mNG forms distinct bright clusters upon ER stress induction by treatment with tunicamycin (Tm). High-magnification confocal microscopy images of these clusters in live cells showed them to be elongated and asymmetric rather than spherical (Fig. 1*B*). Closer inspection revealed other interesting topologies, such as undulating curves and branches. This prompted us to obtain higher-resolution images using structured illumination superresolution microscopy (SIM). Deconvoluted SIM images of IRE1-mNG clusters in fixed MEFs-IRE1-mNG cells with and without transient transfection with the ER marker HaloTag-Sec61β (35) (Fig. 1 *C* and *D* and *SI Appendix*, Fig. S2) revealed a broad range of 3D structures. Many large IRE1 clusters comprised multiple closed loops and branch points, while some smaller clusters had a ring-like appearance. We did not observe any apparent alterations in overall ER organization in or around IRE1 clusters. While every IRE1 cluster showed at least partial 3D overlap with the ER network, we frequently observed an apparent exclusion of Sec61β from small regions within each single cluster (Fig. 1*D*). The previously unrecognized complexity and unexpected structural diversity of IRE1 clusters further highlighted the outstanding questions of how IRE1 molecules are packed and how they exchange into and out of clusters over the progression of ER stress.

### Asymmetric Formation and Dissolution Dynamics of IRE1 Clusters.

**Fig. 2.**
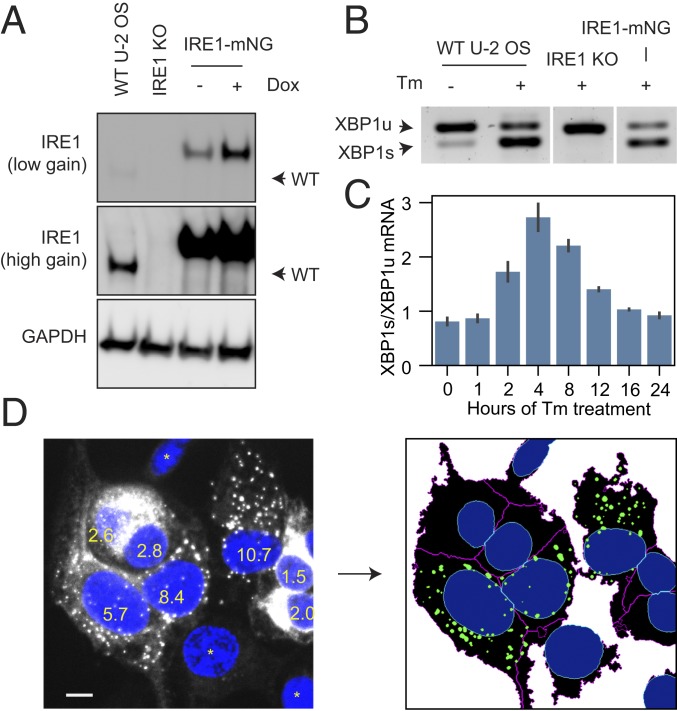
Reconstitution and quantification of IRE1 clustering. (*A*) Immunoblot against IRE1α in parental U-2 OS Flp-In T-Rex cells, IRE1α_KO_ cells, and IRE1-mNG cells expressing IRE1-mNG under a doxycycline-inducible promoter. (*B*) Stress-dependent splicing of *XBP1* mRNA in parental, IRE1α_KO_, and IRE1-mNG cells treated with 5 μg/mL Tm for 2 h where indicated, assessed by RT-PCR. (*C*) Quantification of the ratio of the *XBP1s* over *XBP1u* mRNA in IRE1-mNG cells over a 24-h stress time course, assessed by densitometry of RT-PCR gels (3 replicates, error bars represent SD) (*D*) Automated detection of IRE1 clustering in microscopy images. Average intensity projections of a confocal *z*-stack of IRE1-mNG cells with DAPI-stained nuclei (*Left*) are filtered, thresholded, and segmented, as described in the text to produce ER, nuclear, and cluster masks (*Right*). Granularity scores with a 1-μm structuring element are superimposed on the nuclei. Asterisks denote cells with low IRE1 expression levels that do not meet the cluster detection threshold. In this dataset, the minimum granularity value that established clustering in the cell was 4.0. (Scale bar, 10 μm.)

We next sought to determine how the morphology of IRE1 clusters evolves in response to prolonged ER stress by quantitative imaging of IRE1 clusters over extended time courses. This required a monoclonal cell line that would support reasonably uniform expression levels of fluorescently tagged IRE1 (a caveat of the polyclonal MEFs-IRE1-mNG cell line), lack any untagged wild-type IRE1, and be morphologically well-suited for microscopy. We used CRISPR-Cas9 to knockout all endogenous alleles of IRE1α in Flp-In T-REx U-2 OS cells (*SI Appendix*, Fig. S3). These IRE1α_KO_ cells produced no detectable IRE1α protein by immunoblot (Fig. 2*A* and *SI Appendix*, Fig. S4) and were unable to splice *XBP1* mRNA upon ER stress induction (Fig. 2*B*). The Flp-In T-REx system then allowed us to reconstitute the IRE1-mNG construct into a single well-defined genomic site, creating an isogenic stable cell line (U2OS-IRE1-mNG). The U2OS-IRE1-mNG cells retained the flat, spread-out morphology of their parental U-2 OS cell line. The reconstituted fluorescent IRE1-mNG correctly localized to the ER, formed structurally complex ER stress-induced clusters (*SI Appendix*, Fig. S5), and rescued the cells’ ability to splice *XBP1* mRNA in a stress-dependent manner (Fig. 2 *B* and *C*).

The clustering of IRE1 has previously been assessed by manual or partially automated analyses of fluorescence microscopy images, an approach that suffers from low throughput and potential researcher bias. To overcome these limitations, we developed a method for robust and rapid identification of clusters across hundreds of images with no human input (*Materials and Methods*). Briefly, we used the IRE1-mNG signal to generate cell ER masks, using DAPI- or Hoechst-stained nuclei as starting points. Then, adaptive local thresholding and granularity-based filtering allowed us to reliably identify IRE1 clusters and assign them to parental cells despite a significant degree of cell-to-cell variability in shape and signal intensity. Visual inspection confirmed that ER masks and IRE1 clusters identified by the algorithm closely matched the expected outcome across a broad range of images (Fig. 2*D*), satisfying the initial requirement for a reproducible, automated, and scalable cluster analysis method.

**Fig. 3.**
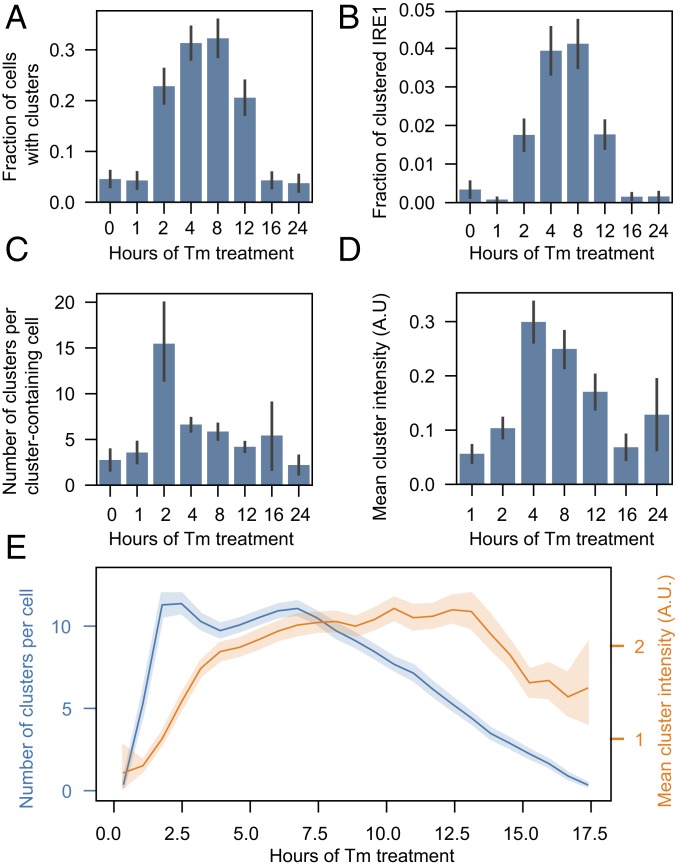
Evolution of IRE1 clusters under sustained ER stress. IRE1-mNG cells were treated with 5 μg/mL Tm for the indicated times, then fixed, imaged, and analyzed as described in the text to plot (*A*) fraction of cells with 1 or more IRE1 clusters, (*B*) fraction of total IRE1 inside clusters, (*C*) number of clusters per cell with 1 or more clusters, and (*D*) cluster intensity. All plots show mean values and 68% CI. Number of cells analyzed for each condition, in ascending order: 178, 164, 149, 207, 177, 146, 163, 134. (*E*) Clustering time course extracted from live imaging of Tm-treated IRE1-mNG cells. Only cells that start off with no clusters at *t* = 0, form multiple clusters upon addition of Tm, and dissolve all clusters by the end of the experiment are used to construct the time course. Number of clusters per cell is plotted in blue and mean cluster intensity is plotted in orange. Data are binned and shown as mean with 95% CI (*n* = 1,601 cells). A.U., arbitrary units.

To quantify cluster evolution as a function of stress duration, cells were treated with 5 μg/mL Tm for periods of time ranging from 1 to 24 h, fixed, and imaged. The number of cells with clusters increased at early time points of stress and decreased at later time points, as reported previously (Fig. 3*A*). Notably, the analysis revealed a striking asymmetry between cluster assembly and disassembly: At early time points of stress, we observed many small clusters that collectively contained a small percentage of each cell’s total pool of IRE1 (Fig. 3 *B* and *C*). As the duration of stress increased, the clusters rapidly grew in intensity but decreased in number (Fig. 3 *C* and *D*). In later time points of stress, the fraction of clustered IRE1 slowly decreased without further changes in number of clusters. By 24 h after Tm addition, all metrics of clustering matched those of the starting baseline. *XBP1* mRNA splicing, quantified as the ratio of *XBP1s* over *XBP1u* (Fig. 2*C*), generally correlated with the average fraction of IRE1 in clusters, gradually increasing at the beginning of stress and decreasing after peaking between 4 and 8 h after Tm addition. We confirmed that the findings are representative of general ER stress rather than a specific effect of Tm by repeating the experiment with an orthogonal ER stress-inducing agent, thapsigargin (*SI Appendix*, Fig. S6).

In this analysis, it became apparent that there exists a large amount of cell-to-cell variation in the number and size of clusters when the cells are observed at fixed time points. This variation might arise either from fundamental differences between the susceptibility of different cells to Tm treatment or from differences in timing of IRE1 clustering between cells. Since fixed-cell experiments preclude us from learning the history or fate of clusters in any given cell, we imaged live cells in a temperature- and CO_2_-controlled incubator for 18 h following addition of Tm. We then used our automated analysis pipeline to identify the Hoechst-stained cell nuclei and the associated ER masks and IRE1 clusters (Movie S1). Imaged at 12 frames per hour, U2OS-IRE1-mNG cells moved slowly enough (in contrast to the more motile MEFs-IRE1-mNG) to allow for easy tracking of individual cells over the entire duration of the experiment.

Analysis of the resulting single-cell trajectories showed that approximately one-half of the cell population never formed detectable clusters over the entire time course of stress. This is not unprecedented, as bifurcation of individual responses in a nominally homogeneous population of cells has been reported in other single-cell imaging experiments, including those measuring Tm-induced *XBP1* splicing (36). We filtered all single-cell trajectories to only include cells in which clusters form and dissolve over the duration of the experiment. Overlaying these filtered trajectories allowed us to construct a typical clustering time course of a Tm-responsive cell (Fig. 3*E*), which again revealed a clear asymmetry between the formation and dissolution of clusters: At the start of the response, the total number of clusters rises rapidly and plateaus at its maximal value, while the mean intensity (and thus size) of clusters increases slowly over the first 3 to 4 h of stress. Then, after a plateau lasting several hours, the number of clusters per cell begins to drop while the mean size of the clusters remain constant until nearly the end of the time course. This indicates that the disappearance of clusters is not synchronized across the population of cells and that every cluster dissipates quickly without breaking up into multiple smaller constituents. Together with the earlier observation that IRE1 clusters can grow by fusion (26), this finding is reminiscent of coalescence and dissolution of liquid droplets (37), leading us to examine the biophysical properties of IRE1 clusters and home in on their assembly and disassembly mechanism.

### Exchange of IRE1 Molecules into and out of Clusters.

**Fig. 4.**
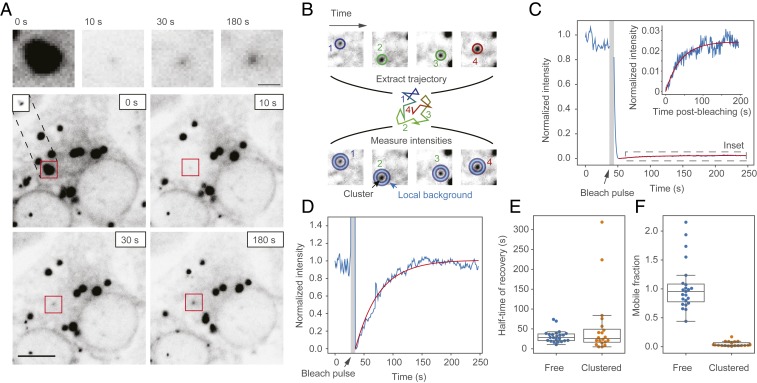
Measuring recovery of IRE1 clusters by FRAP. (*A*) Representative image of an IRE1-mNG cell where 1 large and 2 small clusters are bleached with a focused 405-nm laser beam. Images at the top are zoomed-in views of the recovering cluster, indicated by the red squares in the images below. *Inset* in the 0-s panel shows the same cluster with lower brightness correction to illustrate that the cluster is nearly diffusion-limited in size and only appears large in the other images due to saturation. All images are γ-corrected with a γ-value of 1.3 to make the faint outline of the ER easier to see. (Scale bars: *Top*, 2 μm; *Bottom*, 10 μm.) (*B*) Schematic depiction of the local background subtraction strategy for monitoring FRAP in recovering clusters. First, the recovering cluster is tracked throughout the movie, yielding a diffusion trajectory (positions and trajectory are colored by frame time). Then, cluster intensity (inner circles, bottom row) is measured and corrected for local background (blue outer circles, bottom row). (*C*) Representative FRAP trace (blue line) and fit (red line) of a bleached IRE1 cluster. *Inset* shows an expanded view of the recovery, which plateaus at ∼3% of the initial intensity and is difficult to see on the full-scale graph. (*D*) Representative FRAP trace of unclustered IRE1 from a 5-μm radius bleached region in a cluster-free region of the ER. (*E*) Comparison of recovery half-time measurements of clustered and unclustered IRE1. (*F*) Comparison of mobile fraction extracted from FRAP curves of clustered and unclustered IRE1.

Other stress-response proteins have been previously found to assemble into phase-separated domains as part of their functional cycle, such as RNA-binding proteins involved in the formation of stress granules and many classes of membrane-bound receptors (31). Since IRE1 contains a long, disordered and low-complexity (38, 39) linker region between its transmembrane and kinase domains and assembles into large supramolecular structures that have been observed to grow by fusion (26), we next asked whether IRE1 clusters exhibit properties characteristic of phase-separated liquid droplets. To this end, we induced ER stress in our U2OS-IRE1-mNG cells and performed fluorescence recovery after photobleaching (FRAP) experiments on individual IRE1-mNG clusters. Fluorescence recovery in completely bleached clusters was evident within seconds of the bleaching pulse (Fig. 4*A*), but the fact that clusters move rapidly over the very nonuniform background of the ER meant that we could not rely on standard fixed-position FRAP analysis for constructing recovery curves. Instead, we tracked the recovering spots over time and performed local background correction in each frame by subtracting the background of a ring centered around each spot from the intensity of the spot itself (Fig. 4*B* and Movie S2). This approach yielded minutes-long FRAP curves that were suitable for further analysis (Fig. 4 *C* and *D*). To establish a reference point, we additionally performed FRAP on nonclustered IRE1-mNG by bleaching a randomly chosen spot within the ER network in regions devoid of clusters.

Our first finding from the nonclustered IRE1-mNG FRAP experiments revealed that individual IRE1 molecules rapidly diffuse throughout the ER network, with fluorescence in a large ∼10-μm diameter bleached area recovering to 50% of its initial fluorescence 30 s after the bleach pulse, and returning to ∼100% of the initial intensity within 2 min of bleaching (Fig. 4*E*). We estimated the diffusion constant of nonclustered IRE1 to be 0.24 ± 0.02 μm^2^/s (SEM), which is lower but within an order-of-magnitude of the 1.3 μm^2^/s value predicted by the Saffman–Delbrück model (40, 41). We posit that most of this discrepancy arises from the fact that IRE1 diffuses along a topologically complex network of 3D membranes rather than the flat membrane sheet assumed by the model. Our data demonstrate that nonclustered IRE1 is not compartmentalized into ER subdomains and can diffuse across cellular length scales on the timescale of tens of seconds to minutes, an order-of-magnitude faster than the onset of *XBP1* mRNA splicing in response to ER stress. This is compatible with models wherein clusters assemble by simple diffusion rather than by active transport of IRE1.

In contrast, FRAP experiments on IRE1-mNG clusters demonstrated that while the characteristic timescale of recovery was statistically indistinguishable (*P* = 0.18, 2-sided *t*-test) from that of nonclustered IRE1, the clusters only recovered to a small fraction (4.6 ± 0.9%, SEM) of their initial intensity (Fig. 4*F*). Our tracking-plus-FRAP approach allowed nearly all FRAP trajectories to reliably reach the plateau phase, meaning that the low percentage of cluster recovery cannot be attributed to insufficient experiment duration or fitting artifacts. This finding draws a sharp distinction between IRE1 and membrane-bound proteins that undergo liquid–liquid de-mixing. For example, the plasma membrane calcium ATPase (PMCA4b) assembles into clustered domains that exchange almost completely with the surrounding membrane when probed by FRAP (42). In contrast, our data suggest that IRE1 clusters contain a comparatively small peripheral region that is free to exchange with the surrounding pool of “free” IRE1 in the ER membrane, and a large central core that is either diffusionally or structurally precluded from such exchange.

### Movement of IRE1 Clusters Is Independent of Their Size.

**Fig. 5.**
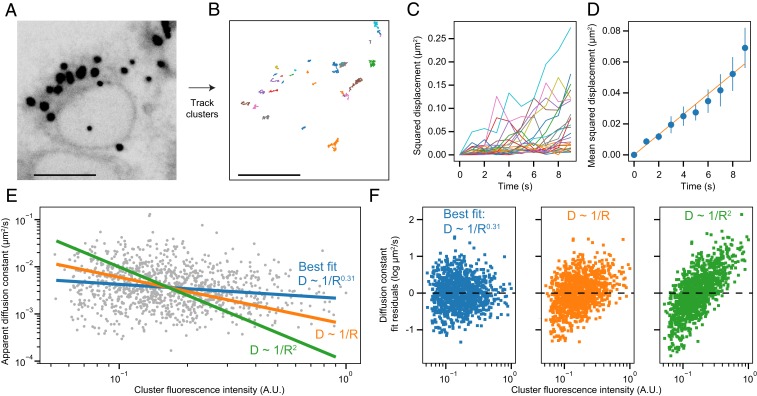
Tracking diffusion of IRE1 clusters. Clusters from a live-imaging experiment (*A*) with a 1-Hz frame rate are tracked to yield single-cluster trajectories (*B*); colors represent individual cluster trajectories. (Scale bars, 10 μm.) The trajectory of each cluster is broken up into individual 9-s chunks (*C*), which are then averaged to provide a separate MSD plot for each cluster (*D*). The MSD plot is fit with a straight line to yield a per-cluster diffusion constant (red line). Values shown are mean and 95% CI. (*E*) Log–log plot of calculated diffusion constants for each cluster plotted against the integrated fluorescence intensity of that cluster, which we take to be directly proportional to the number of IRE1 molecules in that cluster or the square root of the cluster’s radius. The best-fit linear regression line (blue) shows a minimal correlation between cluster intensity and diffusion constant, while enforcing a 1/*R* (orange) or 1/*R*^2^ (green) dependence results in substantially worse fits. (*F*) Residual plots of the 3 fits in *E*. A.U., arbitrary units.

The pronounced movement of IRE1 clusters that necessitated tracking them in FRAP experiments prompted a more thorough analysis of their motility. The ER is known to extensively contact many organelles, and IRE1 has been postulated to play a role in such contacts (e.g., by connecting the ER to the cytoskeleton or to mitochondria) (29, 30). We wondered whether IRE1 clusters are actively trafficked or tethered to cellular structures that may in turn regulate their assembly or disassembly. To test this notion, we checked whether the movement of clusters within the ER network would be consistent with that of a freely diffusing membrane-bound inclusion or with that of a structure constrained by additional interactions. We tracked ∼4,500 IRE1-mNG clusters in live cells (Fig. 5 *A* and *B*), binned each trajectory into 10-s chunks (Fig. 5*C*), and constructed individual mean squared displacement (MSD) vs. time plots for each cluster (Fig. 5*D*). MSD increased linearly with time for IRE1 clusters, suggesting predominantly diffusive motion rather than constrained diffusion or active transport. When we extracted diffusion constants from the MSD plots of individual clusters, the values were generally 2 orders-of-magnitude lower than those estimated for nonclustered IRE1 in our FRAP experiments. However, we were intrigued to observe that the apparent diffusion constant is nearly independent from cluster radius, which we assume to be proportional to the square root of the cluster’s integrated fluorescence intensity.

Several models have been proposed for the diffusion of large membrane inclusions, with different assumptions leading to different dependencies of the diffusion constant *D* on the inclusion’s radius *R*. Treating the inclusion as a rigid cylinder results in an inverse linear scaling of *D* ∼ 1/*R* (43), while allowing for internal diffusion within the cluster yields a steeper dependence of *D* ∼ 1/*R*^2^ (41). However, our data fit poorly with both a *D* ∼ 1/*R* and *D* ∼ 1/*R*^2^ model (with *R*^2^ values of −0.13 and −0.89, respectively), instead exhibiting a very weak size-dependence of *D* ∼ 1/*R*^0.3^ (Fig. 5 *E* and *F*) with a correlation coefficient of −0.18 (95% CI [−0.23, −0.12]). This calculation suggests 2 exciting possibilities: Either 1) IRE1 clusters diffuse in complex with large subcellular structures that are invisible in our experiment, or 2) the arrangement of IRE1 molecules within a cluster confers a preference for a particular subdomain of the ER such that the cluster moves together with the ER membrane rather than diffusing through it. In either scenario, we can conclude that IRE1 clusters are not directly trafficked along cytoskeletal filaments, but likely form mechanical contacts with other ER-adjacent entities.

### Tracking the Fate of IRE1 Molecules from the Diffusionally Trapped Core.

**Fig. 6.**
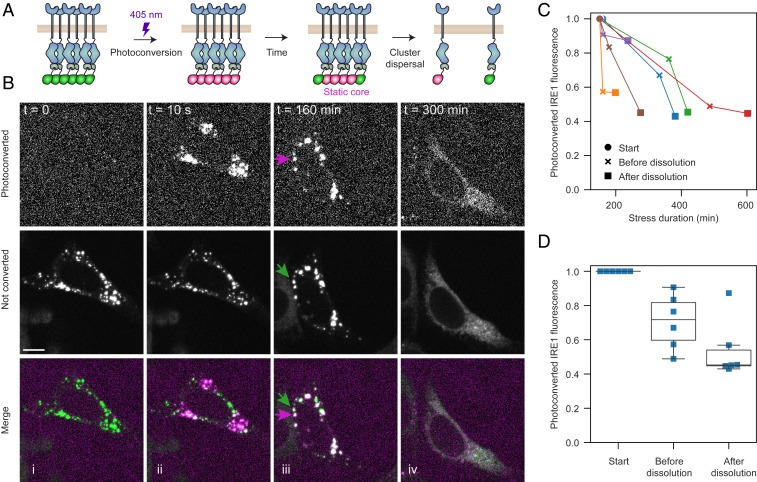
Following the fate of clustered IRE1. (*A*) Schematic depiction of the construct and experiment. IRE1 tagged with a C-terminal mEos4b is expressed in U-2 OS IRE1α_KO_ cells. When cells are treated with 5 μg/mL Tm, IRE1 assembles into large clusters. A single pulse of a focused 405-nm laser irreversibly converts the mEos4b in a cluster from green to red. We then follow the fate of the photoconverted clusters until their dissolution. (*B*) Representative frames from a live-cell imaging experiment. Before photoconversion, all clusters fluoresce in the 488-nm channel (represented in green; *Middle* row). Immediately after, we photoconvert 3 distinct sets of clusters, they appear in the 561-nm channel (represented in magenta; *Top* row). Hours after photoconversion, the red and green clusters remain largely demixed (predominantly red and green clusters shown with magenta and green arrows). Finally, when clusters dissolve, both red and green fluorescence is redistributed throughout the ER network. (Scale bar, 10 μm.) (*C*) Plot of the total photoconverted intensity (measured in the 561 channel) from *n* = 6 cells, with each line representing a different cell. For each cell, the 3 time points shown are immediately after photoconversion (solid circles), the last frame before the beginning of detectable cluster dissolution (*x* marks), and the first frame after the disappearance of the last detectable cluster (solid squares). (*D*) Quantification of the fraction of remaining photoconverted IRE1 fluorescence at each of the 3 time-points plotted in *C*.

If the majority of IRE1 molecules within a given cluster are locked in a central core, how do clusters dissolve in response to prolonged ER stress? One possibility is that the trapping mechanism is released by a regulatory switch that is triggered by up-regulation of ER-lumenal chaperones (44), activation of an IRE1-specific phosphatase (45), or other means. This possibility has been suggested in the past but has not been explicitly tested. Alternatively, IRE1 clusters could be specifically targeted by protein turnover machineries (46) or eventually recognized by the cell as insoluble aggregates marked for autophagic degradation. To directly visualize the fate of clustered IRE1, we tagged IRE1 (at the C terminus) with the photoconvertible protein mEos4b, which undergoes an irreversible chemical change upon illumination with near-UV light that shifts its emission spectrum from green to red (Fig. 6*A*). We integrated IRE1-mEos4b into the FRT locus of our U-2 OS IRE1α_KO_ cells and confirmed that IRE1-mEos4b correctly localized to the ER and formed clusters in response to Tm-induced ER stress. We then photoconverted individual IRE1 clusters in live cells with a focused 405-nm laser beam and imaged the cells in both red and green channels until clusters dissolved (Fig. 6*B* and Movie S3).

First, the photoconversion experiment satisfyingly confirmed our conclusion from FRAP experiments that IRE1 clusters contain a large immobile core: Hours after conversion, the converted clusters remained predominantly red and the nonconverted clusters remained green (140 of 155 observed clusters retaining their original color), with the notable exception of cluster fusion events that yielded larger clusters with both red and green fluorescence. Due to the small, nearly diffraction-limited size of clusters and poor photostability of the mEos4b fluorophore, we could not resolve whether the cores of the merging clusters intermix or merely join together; however, due to the noncircular shape of large late-stage clusters in our earlier fixed-cell experiments (Fig. 1*C*), we favor the latter possibility.

Imaging the dissolution of photoconverted clusters provided unequivocal evidence that the trapped IRE1 molecules are indeed released back into the ER network rather than degraded. In all cells that remained in the field-of-view for the duration of experiment and in which the levels of photoconverted IRE1 did not fall below a detectable threshold, about three-fourths (77.4 ± 8.3%, SEM) of red fluorescence intensity that was previously contained in clusters returned to a diffuse ER-like distribution (Fig. 6 *C* and *D*). The three-fourths estimate serves as a conservative lower-bound, since we found the majority of photoconverted mEos4b signal loss to be independent of ER stress and caused by photobleaching (*SI Appendix*, Fig. S7). Since our earlier experiments show that the size of IRE1 clusters remains invariant throughout the majority of the stress time course, their seemingly sudden and rapid dispersal bears the hallmarks of a regulatory switch that overrides IRE1’s propensity for clustering.

## Discussion

The mRNA splicing activity of IRE1 has been extensively correlated with its oligomerization and microscopically visible clustering; yet, the nature of these clusters has remained largely unexplored. While IRE1’s lumenal and cytosolic domains separately exhibit a propensity for oligomerization (14, 17), thorough biophysical analyses of full-length IRE1 in vitro are hindered by challenges in purifying and reconstituting the full-length and membrane-embedded protein. By analyzing IRE1 clustering in living human and mouse cells, we uncovered a number of surprising properties of the clusters. First, in contrast to qualitative impressions gleaned from microscopic images, IRE1 clusters comprise only a small fraction (∼5%) of the total IRE1 in the cell. Second, IRE1 clusters have complex topologies indicating that they are not simply 2D patches, as previously proposed, but display features of higher-order organization. Third, IRE1 clusters are not phase-separated liquid condensates and instead contain a diffusionally constrained core. Fourth, IRE1 clusters remain diffusionally accessible to the free pool of IRE1 in the general ER network. Fifth, when IRE1 clusters disappear at later time points of stress as IRE1 signaling attenuates, their constituent molecules are released into the ER network rather than degraded. Sixth, IRE1 cluster assembly and disassembly are mechanistically distinct. Finally, IRE1 clusters’ mobility is independent of cluster sizes, which is most easily explained by their tethering to larger cellular structures such as the cytoskeleton. Taken together, these insights accentuate the importance of studying IRE1 dynamics in native ER membranes.

Our findings allow us to reconstruct the life cycle of IRE1 clusters in a cell: Within tens of minutes of the onset of acute ER stress, between 10 and 100 clusters are concomitantly nucleated throughout the ER network. Initially small, these clusters grow by lateral diffusion coupled with incorporation of additional IRE1 molecules and by coalescence of clusters until their size reaches a surprisingly stable plateau. It remains unclear how additional cluster–cluster fusions are curtailed during the plateau phase. At no point do IRE1 clusters lose continuity with the ER membrane, but the majority of IRE1 molecules remain trapped inside the clusters. Trapping suggests that clusters consist of 2 distinct pools of IRE1, of which only a small pool (about 5%) remain exchangeable. This finding parallels our observation that many large IRE1 clusters contain spatially distinct regions devoid of the ER membrane protein Sec61β. Trapping of clustered IRE1 could either be by diffusional restriction—for example, if the exchanging and trapped pools of IRE1 are physically separated by a diffusion barrier—or by structural restriction: for example, if IRE1 molecules assemble into highly ordered structures that can only gain or lose new subunits at exposed edges.

As stress continues unabated for several hours, the plateau phase comes to an end. IRE1 signaling attenuates, and IRE1 clusters begin to melt back into the ER. Intriguingly, IRE1 clusters do not break up into many smaller constituents before disappearing. While this observation resembles the dissolution of liquid–liquid droplets, our finding that clusters contain a large population of nonexchanging IRE1 refutes the notion that clusters are amorphous phase-separated condensates. Their abrupt dissolution bears the hallmarks of an externally regulated process as previously shown. Two known contributors to IRE1 attenuation are the dephosphorylation of clustered IRE1 via PKR-like ER kinase (PERK)-dependent RNA polymerase II associated protein (RPAP2) synthesis on the cytosolic face (45), and the binding of the ER chaperone ERdj4 on the lumenal face of the ER (44). The relative contributions of these 2 molecular players to the dispersal of IRE1 clusters, as well as the contributions of any additional yet-unknown factors, remain to be determined.

IRE1 is a low-abundance protein, which we overexpress by approximately a factor of 10 compared to endogenous levels. Overexpression was required as a necessity for the microscopy experiments, yet imposes the caveat that IRE1 clustering may arise as a byproduct of overexpression. However, this expression level corresponds to ∼100,000 molecules in an entire cell or roughly estimated to be on the order of 10 molecules per square micrometer of ER membrane (see *Materials and Methods* for the assumptions made in this estimation). As this calculation indicates, even at the overexpression level used here IRE1 remains sparsely distributed in the ER membrane. Moreover, others have expressed IRE1 at levels as low as 2× over endogenous protein (26) and still observed robust stress-dependent clustering. A second potential caveat arises from the tagging of IRE1 with fluorescent proteins (FPs). However, IRE1-FP clustering did not occur in control cells but strictly remained responsive to ER stress. Moreover, this finding was reproduced in IRE1 constructs fused to EGFP (25), as well as highly monomeric mNG and mEos4b (present work). Furthermore, clustering occurred both when the FP fusion was located internally in the flexible cytoplasmic linker, as in our IRE1-mNG construct, or C terminally, as in IRE1-mEos4b.

Currently, the role of IRE1 clusters remains an enigma. As previously suggested, IRE1 clusters can be thought of as “splicing factories” for concentrating highly oligomerized and hyperactivated IRE1. Clusters could also serve as temporary storage compartments for sequestering excessive IRE1 and buffering against overactivation of the pathway. Yet another possibility is that clustered and “free” IRE1 molecules act on distinct mRNA substrates via selective targeting of either *XBP1* mRNA or regulated IRE1-dependent decay substrates. Finally, IRE1 clusters could serve as scaffolds for assembly of additional signaling moieties that transmit information independent of IRE1’s RNase activity (47). The tools developed here will be instrumental in distinguishing between these exciting possibilities and open similarly quantitative investigations of other subcellular dynamic assemblies.

## Materials and Methods

### Cell Culture and Experimental Reagents.

U-2 OS Flp-In T-REx cells were a kind gift of the Ivan Dikic laboratory, Institute of Biochemistry II, School of Medicine, Goethe University, Frankfurt am Main, Germany, and were independently authenticated through the human STR profiling service offered by the American Type Culture Collection (ATCC). Cells were cultured in high-glucose DMEM (Thermo Fisher) supplemented with 10% tetracycline-free FBS (Takara Bio), 6 mM l-glutamine, and 100 U/mL penicillin/streptomycin. All cell lines used in the study tested negative for mycoplasma contamination when assayed with the Universal *Mycoplasma* Detection Kit (ATCC 30-1012K). Tm was purchased from Sigma. Halo-Sec61-C-18 was a kind gift from Kevin McGowan, Janelia Research Campus, Howard Hughes Medical Institute, Ashburn, VA (Addgene plasmid #123285; http://www.addgene.org/123285/; RRID:Addgene_123285). HaloTag-JF_549_ was a kind gift from Luke Lavis, Janelia Research Campus, Howard Hughes Medical Institute, Ashburn, VA. The following antibodies were used: rabbit anti-IRE1α (14C10; Cell Signaling 3294S), rabbit anti-GAPDH (Abcam ab9485), and HRP-conjugated donkey anti-rabbit (GE Healthcare NA9340V).

### Generation of IRE1α KO U-2 OS Cell Line.

To generate the IRE1α KO cell line, we assembled and introduced recombinant Cas9–single-guide RNA (sgRNA) ribonucleoprotein (RNP) complexes into U-2 OS Flp-In T-REx cells as described previously (48, 49). First, we chose 10 candidate guide RNA sequences targeting regions in the first 3 exons of the IRE1α (ERN1) gene using the CRISPR guide design tool provided by Benchling (https://www.benchling.com/crispr/). Each guide sequence was assembled into the a final sgRNA template by PCR, with the final template having the following form: *TAATACGACTCACTATAGG***TCCTCGCCATGCCGGCCCGG**GTTTAAGAGCTATGCTGGAAACAGCATAGCAAGTTTAAATAAGGCTAGTCCGTTATCAACTTGAAAAAGTGGCACCGAGTCGGTGCTTTTTTT, where the T7 polymerase promoter is italicized, the variable 20-nucleotide sgRNA is shown in bold and underlined, and the remaining nucleotides comprise the sgRNA constant region. The sgRNA sequence shown above was our best-performing guide out of the 10 guides tested and targeted the region immediately adjacent to the start codon of ERN1. The assembled sgRNA templates were in vitro-transcribed with T7 RNA polymerase (New England Biolabs HiScribe E2040), treated with DNase (Thermo AM2238), purified using Zymo RNA Clean & Concentrator-5 columns (Zymo R1016), and stored at −80 °C.

The Cas9–sgRNA RNP complexes were assembled immediately prior to nucleofection. A 130-pmol quantity of sgRNA was diluted in Cas9 buffer (150 mM KCl, 20 mM Tris pH 7.5, 10% [vol/vol] glycerol, 1 mM TCEP-HCl, 1 mM MgCl_2_, all RNase-free in DEPC-treated H_2_O) and incubated at 70 °C for 5 min to refold the RNA. Then, the sgRNA was mixed with 100 pmol of purified Cas9 protein (kind gift of the Jonathan Weissman laboratory, Department of Cellular and Molecular Pharmacology and Howard Hughes Medical Institute, University of California, San Francisco, CA) in a final reaction volume of 10 μL and incubated at 37 °C for 10 min. Then, 200,000 U-2 OS Flp-In T-REx cells were electroporated with the 10 μL Cas9–sgRNA mixture on an Amaxa Nucleofector and plated into wells of a 24-well plate for recovery. Four days after nucleofection, half of the cells were harvested for the isolation of genomic DNA (gDNA) using the PureLink Genomic DNA Mini Kit (Thermo Fisher). The edited region was amplified by PCR (forward primer sequence: AGCGCTTATAGGGCCGGGAA; reverse primer sequence: GTTCAAACAAGGATTCGAAGCGCAGG) and the ∼600-bp amplicons were tested for cleavage efficiency with a T7 endonuclease I mismatch assay. In this assay, the amplicons are heated to 95 °C for 10 min, then annealed in 7 steps of decreasing temperature. Each step lasted for 1 min and decreased the temperature by 10 °C. The reannealed amplicons were then incubated with T7 Endonuclease 1 (New England Biolabs M0302) for 1 h at 37 °C and resolved by agarose gel electrophoresis. Based on the results of the mismatch assay, we focused on 2 sgRNA sequences that yielded the highest efficiency of editing and split these cells into single clones by limiting dilution.

After the single clones were expanded, their gDNA was extracted and assayed for editing using the T7 Endonuclease assay as described above. For the clones that showed the best results in the endonuclease assay, the ∼600-bp amplicons were gel-purified and cloned into bacterial vectors for sequencing (Zero Blunt TOPO PCR cloning kit, Thermo Fisher). At least 15 bacterial colonies were sequenced for each clone. Of the ∼40 clones that were initially analyzed with the t7 endonuclease assay, 1 had frame-shift or start codon deletion mutations in each allele of ERN1. This clone was confirmed to be a complete functional knock-out of IRE1 by Western blotting and *XBP1* RNA cleavage assays.

### Reconstitution of IRE1α Expression in KO Cells.

The Flp-In system was used to reconstitute fluorescently labeled IRE1 into the IRE1 KO U-2 OS cell line. Parental cells were plated onto wells of a 6-well plate at a density of 1.7E4 cells/cm^2^. The following day, they were cotransfected with 1.7 μg of the Flp recombinase expression vector, pOG44 (Thermo Fisher V600520), and 300 ng of tagged IRE1 incorporated into the pcDNA5/FRT/TO backbone (Thermo Fisher V652020). Transfections were carried out in antibiotic-free DMEM, using the Fugene HD transfection reagent (Promega E2311) and following the manufacturer’s protocol. Twenty-four hours after transfection, cells were split 1:6 into 10-cm dishes and given another 24 h to adhere and recover. The growth medium was then supplemented with 150 μg/mL hygromycin B (Thermo Fisher 10687010) to initiate selection. Cells were maintained in selection medium until single clones became clearly visible and reached ∼3 mm in diameter. Between 3 and 6 clones per cell line were picked using sterile cloning cylinders (Corning 3166-10), expanded, and assayed for doxycycline-inducible expression of the recombinant construct.

To recombinantly express fluorescently tagged IRE1 in IRE1 double-KO MEFs (IRE1α^−/−^/IRE1β^−/−^, kind gift of David Ron, University of Cambridge, United Kingdom), we followed the same strategy as described previously (25). Briefly, the IRE1-mNG coding sequence was introduced into the Gateway entry vector pSHUTTLE-CMV-TO (kind gift of Avi Ashkenazi, Cancer Immunology, Genentech, South San Francisco, CA) (50). The resulting clones were recombined into pGpHUSH.puro (kind gift of Avi Ashkenazi) (50), a single lentivirus expression vector that enables doxycyline-regulatable expression of a gene of interest. Vesicular stomatitis virus (VSV)-G pseudotyped lentiviral particles were prepared using standard protocols using 293METR packaging cells (kind gift of Brian Ravinovich, formerly at MD Anderson Cancer Center, Camden, NJ) (51). Viral supernatants were concentrated by filtration (Amicon Ultra centrifugal filter device, 100-kDa molecular mass cutoff) and used to infect target cells by centrifugal inoculation at 2,000 rpm in a Beckman GH3.8 rotor outfitted with plate carriers for 90 min in presence of 8 μg/mL polybrene. The cells were left to recover overnight following infection and were then subjected to puromycin selection followed by FACS to isolate a polyclonal population expressing IRE1-mNG.

### Estimation of IRE1 Membrane Density.

We estimated that a WT U-2 OS cell contains ∼10,000 copies of IRE1. In our cell lines, IRE1 was overexpressed by a factor of 10 as revealed by Western blot densitometry, using purified truncated IRE1 (25) as a control, yielding ∼100,000 copies of IRE1-mNG or IRE1-mEos4b per cell. Based on microscopy images, we modeled a typical adherent U-2 OS cell as a nearly flat disk roughly 25 μm in diameter, giving the cell a total plasma membrane area of 2 × π × (12.5 μm)^2^ ∼ 1,000 μm^2^. Assuming that a typical cell contains ∼10 times more ER membrane than plasma membrane by area gives us an ER membrane area estimate of 10,000 μm^2^, which results in our final estimate of 100,000 IRE1 molecules/10,000 μm^2^ membrane ∼ 10 IRE1 molecules per square micrometer of ER membrane.

### *XBP1* mRNA Splicing Assays.

Adherent cells were grown in wells of a 12-well plate, treated with doxycycline or ER stressors, and harvested at ∼70% confluency with TRIzol (Thermo Fisher) in accordance with the manufacturer’s instructions. RNA was then extracted from the aqueous phase using a spin column-based purification kit (RNA Clean & Concentrator-5, Zymo Research # R1015) and reverse-transcribed into cDNA using SuperScript VILO Master Mix (Thermo Fisher # 11755050). The cDNA was diluted 1:10 and used as a template for PCR with the following primer pair: VB_pr259_HsXBP1_L (CGGAAGCCAAGGGGAATGAA) and VB_pr167_HsXBP1_R (ACTGGGTCCAAGTTGTCCAG). PCR was carried out with Taq polymerase (Thermo Fisher # 10342020) in the manufacturer-supplied Taq buffer supplemented with 1.5 μM Mg^2+^. The following PCR program was used: 1) Initial denaturation: 95 °C for 2 min; 2) 95 °C for 30 s; 3) 60 °C for 30 s; 4) 72 °C at 30 s; 5) repeat steps 2 to 4 27 more times, for 28 total PCR cycles. PCR products were visualized on a 3% agarose gel stained with SYBR Safe (Thermo Fisher S33102) and imaged on a Typhoon gel imager (GE Healthcare). To obtain the ratio of *XBP1s* to *XBP1u* mRNA, the intensity of each band was independently measured and corrected for local background in ImageJ.

### Western Blotting.

Approximately 200,000 cells were harvested by trypsinization at ∼70% confluency, washed twice with warm PBS, and centrifuged for 7 min at 3,000 relative centrifugal force in a 1.5-mL microcentrifuge tube. The buffer was then aspirated and the cell pellet was resuspended in 80 μL of freshly prepared lysis buffer (30% glycerol, 200 mM Tris, 10% SDS, 0.01% bromophenol blue, 40 mM DTT, 1× cOmplete protease inhibitor mixture [Roche 11 873 580 001], pH 8.0). The resuspended pellet was incubated on ice for 15 min, vortexed at 4 °C for 10 min, then incubated on ice for an additional 10 min. The lysate was then boiled for 5 min and loaded onto an Any kDa Denaturing Gel (Bio-Rad). After electrophoresis (180 V, 50 min, room temperature), proteins were transferred onto a nitrocellulose membrane. The membrane was rinsed briefly with water, blocked with 5% fat-free milk in TBST (20 mM Tris, 150 mM NaCl, 0.1% Tween 20, pH 7.6) for 1 h at room temperature, then cut and incubated with primary antibodies in 5% milk/TBST overnight at 4 °C with agitation. The following day, membranes were washed 3× with TBST, incubated with the secondary antibodies in 5% milk/TBST for 1 h at room temperature, washed 3× with TBST, and developed with SuperSignal West Femto (Thermo Fisher #34095). Developed membranes were imaged on a LI-COR Odyssey gel imager for 30 min.

### Sample Preparation for Microscopy.

Unless indicated otherwise, cells were grown on glass-bottom 35-mm dishes (MatTek) or glass-bottom 8-well chamber slide (Ibidi 80827) coated with Superfibronectin (Sigma-Aldrich S5171) at 5 μg/mL for MEF cell lines or rat tail collagen type I (Corning 354236) at 10 μg/cm^2^ for U-2 OS cell lines. Samples for live-cell imaging experiments were grown in FluroBrite DMEM (ThermoFisher) supplemented with 10% tetracycline-free FBS (Takara Bio), 6 mM l-glutamine, and 100 U/mL penicillin/streptomycin. For fixed-cell imaging, cells were fixed with cold methanol for 4 min at −30 °C followed by DAPI staining and 4 washes with PHEM buffer (60 mM Pipes, 25 mM Hepes, 10 mM EGTA, 2mM MgCl_2_, pH 6.9). Fixed samples were stored and imaged in PHEM buffer.

### Microscopy.

All live-cell and fixed-cell confocal imaging was carried out on a Nikon Ti-E inverted microscope equipped with a Yokogawa CSU-X high-speed confocal scanner unit and a pair of Andor iXon 512 × 512 EMCCD cameras. High-magnification images were obtained using a 100× 1.49 NA oil-immersion objective, while lower magnification images for high-content imaging were acquired through a 40× 1.3 NA oil immersion objective. Images were typically acquired with 50× EM gain and 100-ms exposure. The 405-nm laser was operated at 10 mW, while 488-mW and 561 lasers were operated at 25 mW. All components of the microscope were controlled by the µManager open source platform (52). The microscope stage was enclosed in a custom-built incubator that maintained preset temperature and CO_2_ levels for prolonged live-imaging experiments. To avoid unintentional selection bias (such as picking cells with higher IRE1 expression levels or more visible IRE1 clusters), fields-of-view were selected by only looking at stained cell nuclei in the 405-nm channel. No cells or fields of view were subsequently excluded from analysis, ensuring that the data faithfully capture the distribution of IRE1 fluorescence across the entire cell population.

### Structured Illumination Microscopy.

Fixed cell 3D-SIM was performed using the DeltaVision OMX SR imaging system (GE Healthcare) outfitted with a 60× 1.42 NA oil-immersion objective lens. Samples were fixed in cold methanol and imaged in PHEM buffer at room temperature using a refractive index number 1.518 immersion oil. The final pixel size is 40 nm, with 125-nm *z*-plane spacing using 3 rotations of the SIM grating. SIM processing was performed using the AcquireSRsoftWoRx acquisition and analysis software, and multicolor images were channel aligned using a matrix generated with Tetraspeck beads.

### Cluster Quantification.

All automated analysis of IRE1 clusters was carried out on *z*-stacks of confocal microscopy images acquired with the 40× oil immersion objective (see *Microscopy*, above, for details on image acquisition). At least 20 randomly chosen fields-of-view were imaged for each condition. First, the *z*-stacks were averaged to create a 2D projection of each field-of-view. The 2D projections were then analyzed in Cell Profiler 3.1.8 (53) using 1 of the 2 provided pipelines (Fig3E_Live_cell_clusters.cppipe for live cells, and Fig3A-D_Fixed_cell_clusters.cppipe for fixed cells). Briefly, the fixed cell pipeline functions as follows: 1) Apply background illumination correction to all images. 2) Locate nuclei by global thresholding in the DAPI channel. 3) Identify ER masks in the mNeonGreen channel by adaptive Otsu thresholding (54) in the IRE1-mNG channel, propagating outwards from the previously identified nuclei. Note that using IRE1-mNG signal for creating general ER masks was a convenient byproduct of the fact that the majority of IRE1 remains freely distributed throughout the ER network even at the peak of clustering. 4) Measure median intensity and granularity (55) for each ER mask with a 1-μm structuring element (a typical apparent size of IRE1 clusters). 5) Identify IRE1 clusters by adaptive Otsu thresholding. 6) Assign IRE1 clusters to “parent” cells based on their spatial overlap with the previously identified ER masks. 7) Filter out misidentified “clusters” from cells that were measured to have low granularity or high median fluorescence intensity in the mNeonGreen channel (both parameters were indicative of cells with abnormally high expression levels that nevertheless do not have clear IRE1 puncta). The live-cell pipeline is essentially the same, except that nuclei are tracked between frames and cells are separated into unique trajectories. The data output from Cell Profiler was parsed and analyzed using the provided Python code. Additional analysis details can be found in the README.md file included with the associated code repository (56).

### FRAP.

FRAP experiments were performed on the same Nikon Ti-E microscope. Cells were imaged at 37 °C in the presence of 5% CO_2_, with 100-ms exposure and 1-Hz frame rate. Selected IRE1 clusters were bleached with a 405-nm, 90-mW focused laser beam steered by a pair of galvo mirrors (Rapp UGA-40) and controlled through µManager. Due to the high concentration of IRE1 in clusters, a 5-s continuous bleaching pulse was required to reliably achieve complete bleaching. After bleaching 1 or a few clusters in a given cell, the cell was continuously imaged for another 200 s. The resulting videos were analyzed as follows. First, a 1-μm diameter region of interest (ROI) was placed on the cluster in the first frame of the video, before the cluster is bleached, using the manual tracking mode of the TrackMate plugin (57) for ImageJ. The cluster was then tracked using TrackMate’s semiautomated tracking mode all of the way until the bleaching frames. In the first few postbleaching frames, before fluorescence intensity began to recover, the ROI was fixed at the last detected position of the cluster. As soon as fluorescence of the cluster began to recover detectably, TrackMate was used to track the cluster in semiautomated mode for at least 50 frames. The included custom ImageJ macro, “Extract_two_radii_TrackMate.ijm”, was then used to extract the intensity value of the cluster itself (an 0.8-μm radius circle around the center of the spot) and the intensity value of the cluster’s local background (a 1.2-μm radius ring surrounding the inner circle) for each frame. This process was repeated for all bleached clusters. Meanwhile, unclustered IRE1 FRAP experiments were performed by selecting and bleaching an arbitrary region of the ER, then manually identifying the bleached spot using the included ImageJ macro “Manual_FRAP_ROI.ijm”. Both clustered and unclustered IRE1 intensity trajectories were analyzed using the provided Python code. Additional analysis details can be found in the README.md file included with the associated code repository (56).

### Cluster Photoconversion.

For photoconversion experiments, cells were imaged at 37 °C in the presence of 5% CO_2_. Cells were pretreated with 5 μg/mL Tm 2.5 h prior to the start of imaging to induce IRE1 clustering. ROIs with 1 or more cells with clearly visible IRE1 clusters were selected after a quick initial scan of the coverslip. One or several clusters in the selected cells were then photoconverted using the same steerable 405-nm laser beam as that used for FRAP experiments, except the laser was operated at 15 mW and a 1-s pulse was sufficient to achieve nearly complete photoconversion. The cells were then imaged overnight at a low frame rate (5-min frame interval) and with 100-ms exposure to minimize photobleaching and phototoxicity. Only cells that did not crawl out of the field-of-view or did not get photobleached past a usable level were selected for subsequent analysis. Whole-cell fluorescence intensities in the photoconverted (561 nm) channel were quantified manually for 4 key frames: 1) Last prephotoconversion frame, 2) first postphotoconversion frame, 3) last frame before IRE1 clusters begin to dissolve, and 4) first frame after clusters had finished dissolving. To obtain these whole-cell intensity values, the 4 key frames were corrected for background illumination, cell masks were manually drawn in ImageJ, and integrated fluorescence intensities in the cell masks were measured. The resulted measurements were then compiled and analyzed using the provided Python code. Additional analysis details can be found in the README.md file included with the associated code repository (56).

### Cluster Tracking.

For IRE1 cluster-tracking experiments, cells expressing IRE1 were pretreated with 5 μg/mL Tm for 2.5 h to induce the formation of clusters and imaged on the spinning-disk confocal microscope at 37 °C in the presence of 5% CO_2_, with 100-ms exposure and 1-Hz frame rate. Clusters were tracked across all frames of the movie using the fully automated tracking mode of the TrackMate plugin for ImageJ and analyzed using the provided Python code. To construct MSD plots for individual clusters, the trajectory of each cluster was broken up into 10-s segments that were then aligned and averaged to obtain the final 10-s-long MSD plot for that cluster. Clusters that were tracked for less than 30 s were excluded from further analysis. Each MSD plot was then fitted to a straight line (<*r*^2^> = 4*Dt*) to determine the respective cluster’s diffusion coefficient. Additional analysis details can be found in the README.md file included with the associated code repository (56).

### Data Availability.

The code used to analyze raw data and generate all figures in this paper is freely available through Zenodo (56). Detailed instructions on running and configuring the code to reproduce the individual figure panels are available in the supplied README.md file. Code is released under the maximally permissive Massachussetts Institute of Technology license. It is functionally divided into IPython notebooks for loading preprocessed data and generating figures, ImageJ scripts for performing simple batch operations on source images, Cell Profiler pipelines for extracting cluster data from images (see *Cluster Quantification*, above, for more details), and source Python files containing data loading and data processing functions used in the IPython notebooks. All raw and processed data are available through Zenodo (58). All cell lines (*SI Appendix*, Table S1) and plasmids (*SI Appendix*, Table S2) used in this paper are available upon request.

## Supplementary Material

Supplementary File

Supplementary File

Supplementary File

Supplementary File

## References

[1] Rutkowski D. T., Kaufman R. J., A trip to the ER: Coping with stress. Trends Cell Biol. 14, 20–28 (2004). 14729177 10.1016/j.tcb.2003.11.001

[2] Kozutsumi Y., Segal M., Normington K., Gething M.-J., Sambrook J., The presence of malfolded proteins in the endoplasmic reticulum signals the induction of glucose-regulated proteins. Nature 332, 462–464 (1988). 3352747 10.1038/332462a0

[3] Ron D., Walter P., Signal integration in the endoplasmic reticulum unfolded protein response. Nat. Rev. Mol. Cell Biol. 8, 519–529 (2007). 17565364 10.1038/nrm2199

[4] Walter P., Ron D., The unfolded protein response: From stress pathway to homeostatic regulation. Science 334, 1081–1086 (2011). 22116877 10.1126/science.1209038

[5] Schuck S., Prinz W. A., Thorn K. S., Voss C., Walter P., Membrane expansion alleviates endoplasmic reticulum stress independently of the unfolded protein response. J. Cell Biol. 187, 525–536 (2009). 19948500 10.1083/jcb.200907074 PMC2779237

[6] Kaufman R. J., Stress signaling from the lumen of the endoplasmic reticulum: Coordination of gene transcriptional and translational controls. Genes Dev. 13, 1211–1233 (1999). 10346810 10.1101/gad.13.10.1211

[7] Cox J. S., Shamu C. E., Walter P., Transcriptional induction of genes encoding endoplasmic reticulum resident proteins requires a transmembrane protein kinase. Cell 73, 1197–1206 (1993). 8513503 10.1016/0092-8674(93)90648-a

[8] Mori K., Ma W., Gething M. J., Sambrook J., A transmembrane protein with a cdc2+/CDC28-related kinase activity is required for signaling from the ER to the nucleus. Cell 74, 743–756 (1993). 8358794 10.1016/0092-8674(93)90521-q

[9] Sidrauski C., Walter P., The transmembrane kinase Ire1p is a site-specific endonuclease that initiates mRNA splicing in the unfolded protein response. Cell 90, 1031–1039 (1997). 9323131 10.1016/s0092-8674(00)80369-4

[10] Tirasophon W., Welihinda A. A., Kaufman R. J., A stress response pathway from the endoplasmic reticulum to the nucleus requires a novel bifunctional protein kinase/endoribonuclease (Ire1p) in mammalian cells. Genes Dev. 12, 1812–1824 (1998). 9637683 10.1101/gad.12.12.1812 PMC316900

[11] Wang X. Z., Cloning of mammalian Ire1 reveals diversity in the ER stress responses. EMBO J. 17, 5708–5717 (1998). 9755171 10.1093/emboj/17.19.5708 PMC1170899

[12] Uhlén M., Proteomics. Tissue-based map of the human proteome. Science 347, 1260419 (2015). 25613900 10.1126/science.1260419

[13] Kimata Y., Two regulatory steps of ER-stress sensor Ire1 involving its cluster formation and interaction with unfolded proteins. J. Cell Biol. 179, 75–86 (2007). 17923530 10.1083/jcb.200704166 PMC2064738

[14] Credle J. J., Finer-Moore J. S., Papa F. R., Stroud R. M., Walter P., On the mechanism of sensing unfolded protein in the endoplasmic reticulum. Proc. Natl. Acad. Sci. U.S.A. 102, 18773–18784 (2005). 16365312 10.1073/pnas.0509487102 PMC1316886

[15] Gardner B. M., Walter P., Unfolded proteins are Ire1-activating ligands that directly induce the unfolded protein response. Science 333, 1891–1894 (2011). 21852455 10.1126/science.1209126 PMC3202989

[16] Ali M. M. U., Structure of the Ire1 autophosphorylation complex and implications for the unfolded protein response. EMBO J. 30, 894–905 (2011). 21317875 10.1038/emboj.2011.18 PMC3049214

[17] Korennykh A. V., The unfolded protein response signals through high-order assembly of Ire1. Nature 457, 687–693 (2009). 19079236 10.1038/nature07661 PMC2846394

[18] Sidrauski C., Cox J. S., Walter P., tRNA ligase is required for regulated mRNA splicing in the unfolded protein response. Cell 87, 405–413 (1996). 8898194 10.1016/s0092-8674(00)81361-6

[19] Cox J. S., Walter P., A novel mechanism for regulating activity of a transcription factor that controls the unfolded protein response. Cell 87, 391–404 (1996). 8898193 10.1016/s0092-8674(00)81360-4

[20] Travers K. J., Functional and genomic analyses reveal an essential coordination between the unfolded protein response and ER-associated degradation. Cell 101, 249–258 (2000). 10847680 10.1016/s0092-8674(00)80835-1

[21] Li H., Korennykh A. V., Behrman S. L., Walter P., Mammalian endoplasmic reticulum stress sensor IRE1 signals by dynamic clustering. Proc. Natl. Acad. Sci. U.S.A. 107, 16113–16118 (2010). 20798350 10.1073/pnas.1010580107 PMC2941319

[22] Korennykh A., Walter P., Structural basis of the unfolded protein response. Annu. Rev. Cell Dev. Biol. 28, 251–277 (2012). 23057742 10.1146/annurev-cellbio-101011-155826

[23] Lee K. P. K., Structure of the dual enzyme Ire1 reveals the basis for catalysis and regulation in nonconventional RNA splicing. Cell 132, 89–100 (2008). 18191223 10.1016/j.cell.2007.10.057 PMC2276645

[24] Sanches M., Structure and mechanism of action of the hydroxy-aryl-aldehyde class of IRE1 endoribonuclease inhibitors. Nat. Commun. 5, 4202 (2014). 25164867 10.1038/ncomms5202 PMC4486471

[25] Karagöz G. E., An unfolded protein-induced conformational switch activates mammalian IRE1. eLife 6, e30700 (2017). 28971800 10.7554/eLife.30700 PMC5699868

[26] Ricci D., Clustering of IRE1α depends on sensing ER stress but not on its RNase activity. FASEB J. 33, 9811–9827 (2019). 31199681 10.1096/fj.201801240RR PMC6704461

[27] Goh L. K., Sorkin A., Endocytosis of receptor tyrosine kinases. Cold Spring Harb. Perspect. Biol. 5, a017459 (2013). 23637288 10.1101/cshperspect.a017459 PMC3632065

[28] Shin Y., Brangwynne C. P., Liquid phase condensation in cell physiology and disease. Science 357, eaaf4382 (2017). 28935776 10.1126/science.aaf4382

[29] Carreras-Sureda A., Non-canonical function of IRE1α determines mitochondria-associated endoplasmic reticulum composition to control calcium transfer and bioenergetics. Nat. Cell Biol. 21, 755–767 (2019). 31110288 10.1038/s41556-019-0329-y PMC7246037

[30] Urra H., IRE1α governs cytoskeleton remodelling and cell migration through a direct interaction with filamin A. Nat. Cell Biol. 20, 942–953 (2018). 30013108 10.1038/s41556-018-0141-0

[31] Chong P. A., Forman-Kay J. D., Liquid-liquid phase separation in cellular signaling systems. Curr. Opin. Struct. Biol. 41, 180–186 (2016). 27552079 10.1016/j.sbi.2016.08.001

[32] Hartman N. C., Groves J. T., Signaling clusters in the cell membrane. Curr. Opin. Cell Biol. 23, 370–376 (2011). 21665455 10.1016/j.ceb.2011.05.003 PMC3703921

[33] Mendez A. S., Endoplasmic reticulum stress-independent activation of unfolded protein response kinases by a small molecule ATP-mimic. eLife 4, e05434 (2015). 10.7554/eLife.05434 PMC4436593 25986605

[34] Shaner N. C., A bright monomeric green fluorescent protein derived from Branchiostoma lanceolatum. Nat. Methods 10, 407–409 (2013). 23524392 10.1038/nmeth.2413 PMC3811051

[35] Grimm J. B., Brown T. A., English B. P., Lionnet T., Lavis L. D., “Synthesis of Janelia fluor HaloTag and SNAP-tag ligands and their use in cellular and imaging experiments” in Methods in Molecular Biology: Super-Resolution Microscopy, Erfle H., Ed. (Humana Press, New York, NY, 2017), pp. 179–188. 10.1007/978-1-4939-7265-4_15 28924668

[36] Walter F., Schmid J., Düssmann H., Concannon C. G., Prehn J. H. M., Imaging of single cell responses to ER stress indicates that the relative dynamics of IRE1/XBP1 and PERK/ATF4 signalling rather than a switch between signalling branches determine cell survival. Cell Death Differ. 22, 1502–1516 (2015). 25633195 10.1038/cdd.2014.241 PMC4532775

[37] Li P., Phase transitions in the assembly of multivalent signalling proteins. Nature 483, 336–340 (2012). 22398450 10.1038/nature10879 PMC3343696

[38] Haerty W., Golding G. B., Low-complexity sequences and single amino acid repeats: Not just “junk” peptide sequences. Genome 53, 753–762 (2010). 20962881 10.1139/g10-063

[39] van Anken E., Specificity in endoplasmic reticulum-stress signaling in yeast entails a step-wise engagement of HAC1 mRNA to clusters of the stress sensor Ire1. eLife 3, e05031 (2014). 25549299 10.7554/eLife.05031 PMC4279078

[40] Saffman P. G., Delbrück M., Brownian motion in biological membranes. Proc. Natl. Acad. Sci. U.S.A. 72, 3111–3113 (1975). 1059096 10.1073/pnas.72.8.3111 PMC432930

[41] Guigas G., Weiss M., Size-dependent diffusion of membrane inclusions. Biophys. J. 91, 2393–2398 (2006). 16829562 10.1529/biophysj.106.087031 PMC1562383

[42] Padányi R., Pászty K., Strehler E. E., Enyedi A., PSD-95 mediates membrane clustering of the human plasma membrane Ca2+ pump isoform 4b. Biochim. Biophys. Acta 1793, 1023–1032 (2009). 19073225 10.1016/j.bbamcr.2008.11.007 PMC2693454

[43] Hughes B. D., Pailthorpe B. A., White L. R., The translational and rotational drag on a cylinder moving in a membrane. J. Fluid Mech. 110, 349–372 (1981).

[44] Amin-Wetzel N., A J-protein co-chaperone recruits BiP to monomerize IRE1 and repress the unfolded protein response. Cell 171, 1625–1637.e13 (2017). 29198525 10.1016/j.cell.2017.10.040 PMC5733394

[45] Chang T.-K., Coordination between two branches of the unfolded protein response determines apoptotic cell fate. Mol. Cell 71, 629–636.e5 (2018). 30118681 10.1016/j.molcel.2018.06.038

[46] Sun S., IRE1α is an endogenous substrate of endoplasmic-reticulum-associated degradation. Nat. Cell Biol. 17, 1546–1555 (2015). 26551274 10.1038/ncb3266 PMC4670240

[47] Hetz C., Glimcher L. H., Fine-tuning of the unfolded protein response: Assembling the IRE1α interactome. Mol. Cell 35, 551–561 (2009). 19748352 10.1016/j.molcel.2009.08.021 PMC3101568

[48] Lin S., Staahl B. T., Alla R. K., Doudna J. A., Enhanced homology-directed human genome engineering by controlled timing of CRISPR/Cas9 delivery. eLife 3, e04766 (2014). 25497837 10.7554/eLife.04766 PMC4383097

[49] Chen B., Dynamic imaging of genomic loci in living human cells by an optimized CRISPR/Cas system. Cell 155, 1479–1491 (2013). 24360272 10.1016/j.cell.2013.12.001 PMC3918502

[50] Gray D. C., pHUSH: A single vector system for conditional gene expression. BMC Biotechnol. 7, 61 (2007). 17897455 10.1186/1472-6750-7-61 PMC2174931

[51] Rabinovich B., NKG2D splice variants: A reexamination of adaptor molecule associations. Immunogenetics 58, 81–88 (2006). 16470377 10.1007/s00251-005-0078-x

[52] Edelstein A., Amodaj N., Hoover K., Vale R., Stuurman N., Computer control of microscopes using μManager. Curr. Protoc. Mol. Biol. 92, 14.20.1–14.20.17. 10.1002/0471142727.mb1420s92 PMC3065365 20890901

[53] McQuin C., CellProfiler 3.0: Next-generation image processing for biology. PLoS Biol. 16, e2005970 (2018). 29969450 10.1371/journal.pbio.2005970 PMC6029841

[54] Otsu N., A threshold selection method from gray-level histograms. IEEE Trans. Syst. Man Cybern. 9, 62–66 (1979).

[55] Vincent L., Granulometries and opening trees. Fundam. Inform. 41, 57–90 (2000).

[56] Belyy V., Analysis code for publication “Quantitative microscopy reveals dynamics and fate of clustered IRE1alpha.” Zenodo. https://zenodo.org/record/3544482. Accessed 16 November 2019.

[57] Tinevez J.-Y., TrackMate: An open and extensible platform for single-particle tracking. Methods 115, 80–90 (2017). 27713081 10.1016/j.ymeth.2016.09.016

[58] Belyy V., Raw and processed data for publication “Quantitative microscopy reveals dynamics and fate of clustered IRE1alpha.” Zenodo. https://zenodo.org/record/3543901. Accessed 16 November 2019.

