# Supplementary material for: Quantitative microscopy reveals dynamics and fate of clustered IRE1α
Source: Proc Natl Acad Sci U S A. 2019 Dec 23;117(3):1533–42. doi: 10.1073/pnas.1915311117 (PMC6983381; doi:10.1073/pnas.1915311117)
Supplement: Supplementary File [file pnas.1915311117.sapp.pdf]

Supplementary Information for

Quantitative microscopy reveals dynamics and fate of clustered IRE1α

Vladislav Belyy*, Ngoc Han Tran*, and Peter Walter#

* Authors contributed equally

#Corresponding author: Peter Walter

Email: Peter@walterlab.ucsf.edu

This PDF file includes:

Figures S1 to S7
Tables S1 to S2
Legends for Movies S1 to S3

www.pnas.org/cgi/doi/10.1073/pnas.1915311117

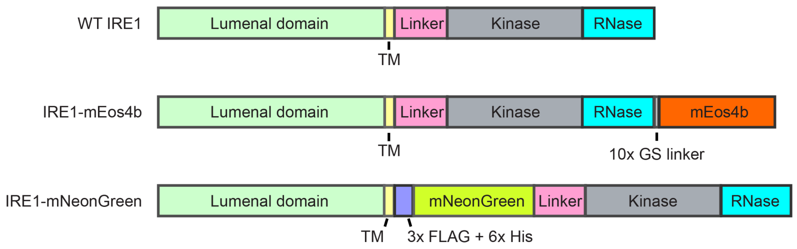

Fig. S1. Genetic maps of all constructs used in this paper**.**

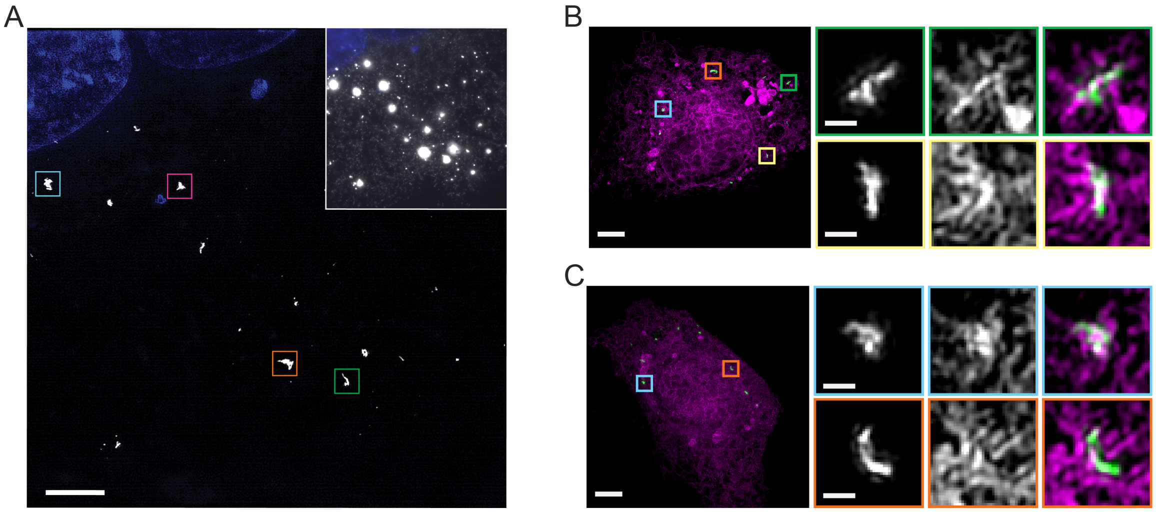

Fig. S2. Maximum intensity projection of deconvoluted Structured Illumination Microscopy (SIM) images of IRE1-mNeonGreen clusters in MEF-IRE1-mNG cells**.** (A) Image of fixed MEF-IRE1-mNG cell at 60X magnification in mNeonGreen and DAPI channels with over-saturated inset. Scale bar: 5 µm. Colored boxes match magnified examples used in Fig. 1C. (B-C) Left: deconvoluted SIM images of stressed (6 hours Tm) and fixed MEF-IRE1-mNG cells transfected with HaloTag-Sec61β and labeled with HaloTag-JF_{549} fluorophore. Scale bar: 5 µm. Right: magnified bilinear interpolation images showing IRE1 cluster structure, local ER structure and overlay image showing IRE1 in green and HaloTag-Sec61β in magenta. Scale bars: 500 nm. Blue and orange boxes in figure B match magnified examples used in Fig. 1D.

```
                                     1        10        20        30        40        50
WT ERN1 exon 1            GAGTCCTCGCCATGCCGGCCCGGCGGCTGCTGCTGCTGCTGACGCTGCTGCTGCCCGGCCTCGGGGTGAGTGACC
sgRNA sequence            ---TCCTCGCCATGCCGGCCCGG----------------------------------------------------
KO allele 1             | GAGTCCTCGCCATGC--------CGGCTGCTGCTGCTGCTGACGCTGCTGCTGCCCGGCCTCGGGGTGAGTGACC
(8 bp deletion)         | GAGTCCTCGCCATGC--------CGGCTGCTGCTGCTGCTGACGCTGCTGCTGCCCGGCCTCGGGGTGAGTGACC
                        | GAGTCCTCGCCATGCCGGC-------------CTGCTGCTGACGCTGCTGCTGCCCGGCCTCGGGGTGAGTGACC
                        | GAGTCCTCGCCATGCCGGC-------------CTGCTGCTGACGCTGCTGCTGCCCGGCCTCGGGGTGAGTGACC
KO allele 2             | GAGTCCTCGCCATGCCGGC-------------CTGCTGCTGACGCTGCTGCTGCCCGGCCTCGGGGTGAGTGACC
(13 bp deletion)        | GAGTCCTCGCCATGCCGGC-------------CTGCTGCTGACGCTGCTGCTGCCCGGCCTCGGGGTGAGTGACC
                        | GAGTCCTCGCCATGCCGGC-------------CTGCTGCTGACGCTGCTGCTGCCCGGCCTCGGGGTGAGTGACC
                        | GAGTCCTCGCCATGCCGGC-------------CTGCTGCTGACGCTGCTGCTGCCCGGCCTCGGGGTGAGTGACC
KO allele 3             | GAGTCCTC--------------------------------------GCTGCTGCCCGGCCTCGGGGTGAGTGACC
(deletion of start codon) | GAGTCCTC--------------------------------------GCTGCTGCCCGGCCTCGGGGTGAGTGACC
```

Fig. S3. Genotyping IRE1 KO cells**.** Sequence alignment showing the individual sequences of three alleles of the IRE1α_{KO} U-2 OS cell line. The coding region of exon 1 of the IRE1 gene (ERN1) is highlighted in light blue. sgRNA sequence used for generating the knockout is shown in green.

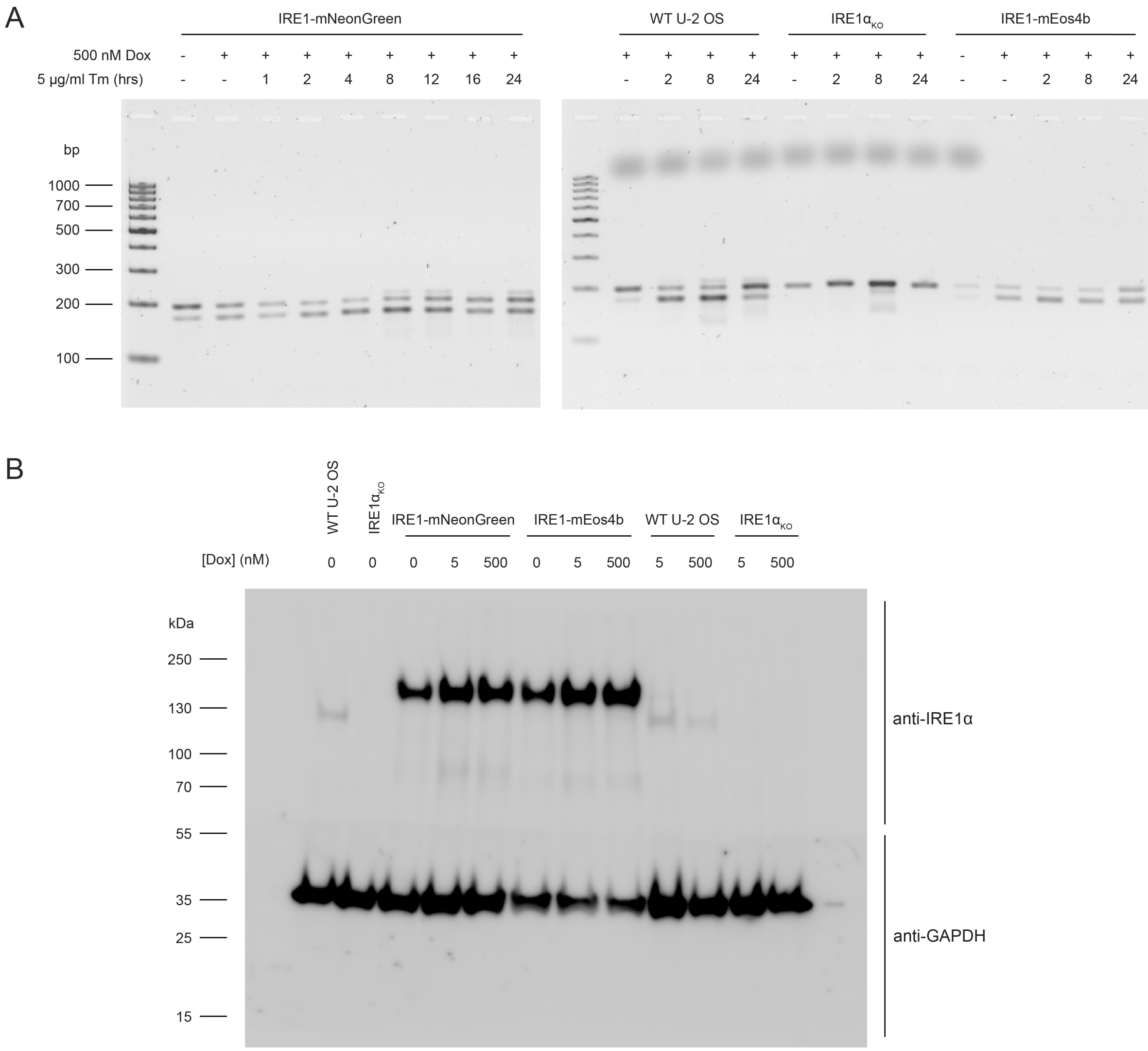

Fig. S4. Uncropped gel images. (A) 3% agarose gel showing *XBP1* mRNA splicing, as determined by RT-PCR. (B) Western blot against IRE1 (top) and GAPDH (bottom) in WT U-2 OS, IRE1α_{KO} U-2 OS, IRE1-mNG, and IRE1-mEos4b cell lines.

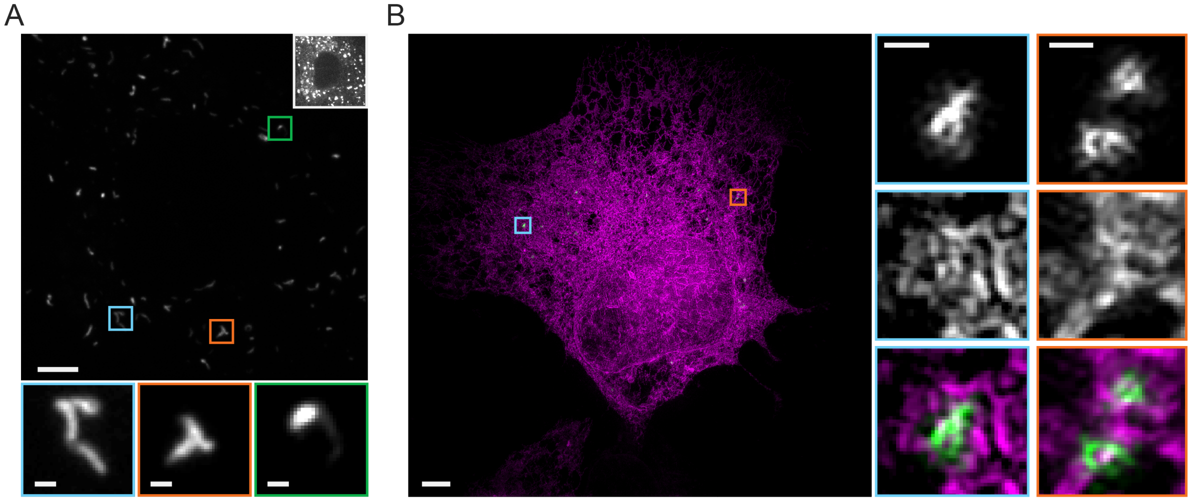

Fig. S5. Diverse morphologies of IRE1 clusters in U2OS-IRE1-mNG cells. (A) Top: maximum intensity projection spinning-disk confocal image of live U2OS-IRE1-mNG cells at 100X magnification in mNeonGreen channel. Over-saturated inset highlights cell shape. Scale bar: 5 µm. Bottom: magnified bilinear interpolation images of interesting cluster morphologies. Scale bars: 500 nm. (B) Left: maximum intensity projection of deconvoluted SIM images of stressed (6 hours Tm) and fixed U2OS-IRE1-mNG cell transfected with HaloTag-Sec61β and labeled with HaloTag-JF$_{549}$ fluorophore. Scale bar: 5 µm. Right: magnified bilinear interpolation images showing IRE1 cluster structure, local ER structure and overlay image showing IRE1 in green and HaloTag-Sec61β in magenta. Scale bars: 500 nm.

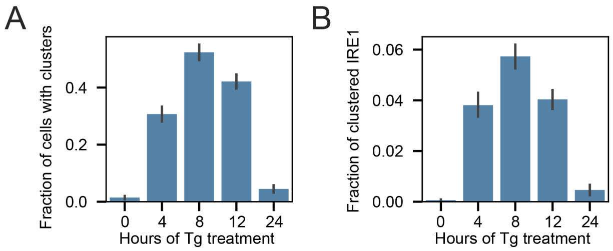

Fig. S6. Analysis of thapsigargin-induced IRE1 clusters. (A) Fraction of cells with clusters at different times of treatment with 1 μM thapsigargin (Tg). (B) Fraction of total IRE1 contained within clusters at different times of Tg treatment.

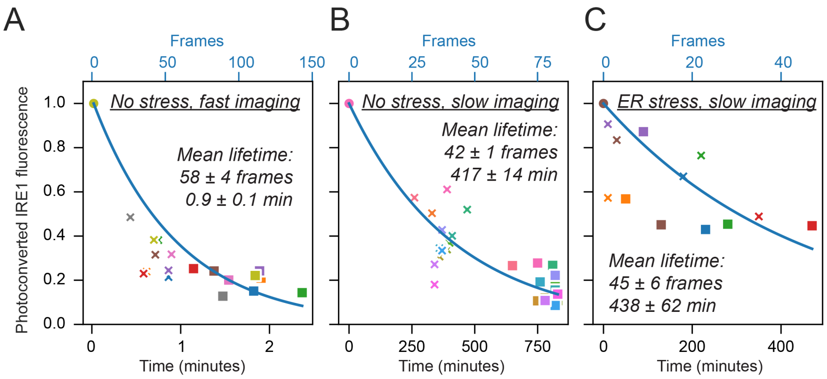

Fig. S7. Photobleaching analysis of photoconverted IRE1-mEos4b. To determine whether the observed disappearance of photoconverted mEos4b signal in Fig. 6 is predominantly caused by protein turnover or photobleaching, IRE1-mEos4b was photoconverted and imaged in unstressed cells. Keeping exposure and laser power unchanged, 'dark' time between subsequent frames was varied from 0.5 s (A) to 10 min (B). Three integrated intensity measurements were taken from each cell: one immediately after photoconversion (denoted by circles), one roughly halfway through the experiment (denoted by x-marks), and one at the end of the experiment (denoted by squares). Marker colors identify individual cells. Disappearance of fluorescence was fitted as a single exponential decay (blue curve; fit results are represented as mean lifetime ± s.d.). Since each frame delivers the same dose of illumination, conservation of mean lifetime in frames but not in minutes indicates that disappearance of fluorescence is predominantly a photobleaching effect. For comparison, data from photoconverted tunicamycin-induced IRE1 clusters are analyzed alongside using the same approach (C).

Table S1. Cell lines used in this study.

Cell Line Name	pWM No.	Organism	Cell type	Culture Type	Antibiotic resistance
MEFs IRE1-mNG-LKR	pWM252	Mouse	fibroblast	adherent	Puromycin
U2OS WT TREX FLP-In	pWM253	Human	osteosarcoma	adherent	Zeocin, blasticidin
U2OS-a2C9 IRE1KO	pWM254	Human	osteosarcoma	adherent	Zeocin, blasticidin
U2OS-IRE1mNG-LKR	pWM255	Human	osteosarcoma	adherent	Hygromycin, blasticidin
U2OS-IRE1-LKR-mEOS	pWM256	Human	osteosarcoma	adherent	Hygromycin, blasticidin

Table S2. Plasmids created for this study.

Plasmid name	Plasmid ID	Backbone	Antibiotic selection	Description
pcDNA5_hIRE1tm3F6H-mNeonGreen-LKR	pPW3444	pcDNA5/FRT/TO	Ampicillin	Human IRE1 with 3x Flag, 6xHis, and mNeonGreen tag in the linker
pShuttle_hIRE1tm3F6H-mNeonGreen-LKR	pPW3445	pShuttle/CMV/TO	Kanamycin	Human IRE1 with 3x Flag, 6xHis, and mNeonGreen tag in the linker
pcDNA5_hIRE1-Cterm-mEos4b	pPW3446	pcDNA5/FRT/TO	Ampicillin	Human IRE1 with 3x Flag + 6xHis in the linker and a C-terminal 10x GS spacer followed by mEos4b

Movie S1. Automated quantification of IRE1 clustering in live-cell data. (Left) Raw imaging data. IRE1-mNG is shown in white and Hoechst-stained nuclei are shown in blue. (Right) Output of the automated image analysis algorithm as described in Methods. ER masks are outlined in magenta, nuclear masks in blue, and IRE1 cluster masks in green.

Movie S2. Tracking a recovering IRE1 cluster in a FRAP experiment. The cluster is bleached at the beginning of the movie and tracked throughout its recovery. The magenta circle indicates the current detected position of the cluster and the cluster's centroid trajectory is shown as a temporally color-coded line.

Movie S3. Following the fate of photoconverted IRE1-mEos4b clusters. This is the full-duration source video from which panels in Figure 6 were chosen. Panels are separated into channels: non-photoconverted 488 nm channel (right), photoconverted 561 nm channel (center), and cyan-red merge of the two channels (left).
